# Novel *Plasmodium falciparum k13* gene polymorphisms from Kisii County, Kenya during an era of artemisinin-based combination therapy deployment

**DOI:** 10.1186/s12936-023-04517-2

**Published:** 2023-03-09

**Authors:** Josephat Nyabayo Maniga, Mong’are Samuel, Odda John, Masai Rael, Jacqueline Njeri Muchiri, Pacifica Bwogo, Odoki Martin, Vidya Sankarapandian, Mfitundinda Wilberforce, Ochweri Albert, Sarah Kemuma Onkoba, Ismail Abiola Adebayo, Rasheed Omotayo Adeyemo, Saheed Adekunle Akinola

**Affiliations:** 1 Department of Medical Microbiology and Immunology, Kampala International University Western Campus, Bushenyi, Uganda; 2 School of Pharmacy, Kampala International University Western Campus, Bushenyi, Uganda; 3 Department of Biological Sciences, Kisii University, Kisii, Kenya; 4 Department of Pharmacology and Therapeutics, Makerere University, Kampala, Uganda; 5 Department of Medical Microbiology and Immunology, School of Medicine, King Ceasor University, Kampala, Uganda; 6 Department of Pharmacology and Toxicology, School of Medicine, King Caesor University, Kampala, Uganda; 7 School of Health Sciences, Kisii University, Kisii, Kenya; 8 Department of Applied Sciences, School of Sciences, Nkumba University, Entebbe, Uganda; 9 Department of Medical Biochemistry, Molecular Biology and Genetics, School of Medicine and Pharmacy, College of Medicine and Health Sciences, University of Rwanda, Butare, Rwanda; 10 Department of Medical Microbiology and Parasitology, School of Medicine and Pharmacy, College of Medicine and Health Sciences, University of Rwanda, Butare, Rwanda

**Keywords:** Artemisinin-based combination therapies, Resistance, *Kelch13* propeller gene, Polymorphism

## Abstract

**Background:**

Currently, chemotherapy stands out as the major malaria intervention strategy, however, anti-malarial resistance may hamper global elimination programs. Artemisinin-based combination therapy (ACT) stands as the drug of choice for the treatment of *Plasmodium falciparum* malaria. *Plasmodium falciparum kelch13* gene mutations are associated with artemisinin resistance. Thus, this study was aimed at evaluating the circulation of *P. falciparum k13* gene polymorphisms from Kisii County, Kenya during an era of ACT deployment.

**Methods:**

Participants suspected to have malaria were recruited. *Plasmodium falciparum* was confirmed using the microscopy method. Malaria-positive patients were treated with artemether-lumefantrine (AL). Blood from participants who tested positive for parasites after day 3 was kept on filter papers. DNA was extracted using chelex-suspension method. A nested polymerase chain reaction (PCR) was conducted and the second-round products were sequenced using the Sanger method. Sequenced products were analysed using DNAsp 5.10.01 software and then blasted on the NCBI for *k13* propeller gene sequence identity using the Basic Local Alignment Search Tool (BLAST). To assess the selection pressure in *P. falciparum* parasite population, Tajima’ D statistic and Fu & Li’s D test in DnaSP software 5.10.01 was used.

**Results:**

Out of 275 enrolled participants, 231 completed the follow-up schedule. 13 (5.6%) had parasites on day 28 hence characterized for recrudescence. Out of the 13 samples suspected of recrudescence, 5 (38%) samples were positively amplified as *P. falciparum,* with polymorphisms in the *k13*-propeller gene detected. Polymorphisms detected in this study includes R539T, N458T, R561H, N431S and A671V, respectively. The sequences have been deposited in NCBI with bio-project number PRJNA885380 and accession numbers SAMN31087434, SAMN31087433, SAMN31087432, SAMN31087431 and SAMN31087430 respectively.

**Conclusions:**

WHO validated polymorphisms in the *k13*-propeller gene previously reported to be associated with ACT resistance were not detected in the *P. falciparum* isolates from Kisii County, Kenya. However, some previously reported un-validated *k13* resistant single nucleotide polymorphisms were reported in this study but with limited occurrences. The study has also reported new SNPs. More studies need to be carried out in the entire country to understand the association of reported mutations if any, with ACT resistance.

## Background

Malaria remains the most prevalent vector-borne tropical disease in the world, causing both mortalities and morbidities, especially in pregnant women and infants. According to the World Health Organization (WHO) in 2021 [1], Kenya registered 6 million malaria cases with 228 million cases reported worldwide, leading to 627,000 deaths globally in the year 2020. This problem is heaviest in sub-Saharan Africa, where approximately 94% of mortalities are registered annually. This situation is predicted to worsen due to the ongoing COVID-19 pandemic, which has greatly compromised malaria treatment and control intervention measures [2].

*Plasmodium falciparum* is the most common parasite, causing about 99% of malaria cases in Kenya [3]. Malaria occurrences in Kenya have variations across the country, with the lake endemic zone having the highest prevalence (27%), followed by the coast endemic zone (8%) and the highland epidemic zone (3%). Kisii County, where this current study was conducted, is located in the Western highland malarial zone. Artemisinin-based combination therapy (ACT) offers highly successful treatment of malaria. However, the emergence and spread of *P. falciparum* parasites with decreased susceptibility to ACT in South-East Asia, South America and some African countries is causing global concern. Artemisinin resistance, defined by slow parasite clearance after treatment with an artemisinin derivative, was first reported in 2007 in Western Cambodia [4].

Timely detection and subsequent monitoring is vital in anticipating actions to contain malaria resistance to ACT in Kenya. Currently, the WHO recommends ACT for the treatment of uncomplicated malaria in most countries. In 2002, Kenya recommended the use of artemether-lumefantrine (AL) as the drug of choice for treating uncomplicated malaria, however, the actual implementation started in 2006 [5]. The increase of anti-malarial-resistant *P. falciparum* has increased malaria deaths globally. Given the increasing reports of resistance or poor responses to ACT in other parts of the world, the sub-Saharan African region affected by the disease may repeat what happened during the emergence of chloroquine and sulfadoxine-pyrimethamine resistance [6]. If such a case arises, malaria control efforts may be compromised. However, with the limited licensed malaria vaccine supply in malaria endemic areas, chemotherapy remains the only option for malaria treatment. Bearing in mind that no new anti-malarial will be available immediately, if ACT fail, this may reverse the significant gains made in the global reduction of malaria over the last 20 years. Mutations in the *kelch13* (*k13*) propeller gene have been used as molecular markers of artemisinin (ART) resistance. Different mutations have been previously reported in Asia, America and Africa continents, with more prevalence recorded in the Asian continent [7]. The evolution and spread of mutant *P. falciparum k13*-mediated artemisinin resistance has led to extensive treatment failure all over the world [8]. Epidemiologically, the frequency of the *k13* mutation is 6.50% in Central Africa, followed by East Africa (5.26%), West Africa (4.55%) and South Africa (4.55%) [9]. Recent reports from Uganda [10, 11] and Rwanda [12] confirmed the presence of ART resistant *P. falciparum*, raising an alarm about the possibility of the same scenario repeating itself in Kenya due to the proximity and frequent border movements between the three East African countries. The circulation of *k13* mutations has also been reported in Kenya but with limited studies [13–15].

To identify the early evolution and spread of *P. falciparum* resistance to ACT the WHO recommends frequent and updated monitoring of their therapeutic efficacy every two years in all malaria-endemic countries. Molecular markers serve as crucial tools for the early detection of drug resistance [16]. Thus, there is an urgent call for continued surveillance of artemisinin resistance markers in Kenya. Therefore, this study sought to establish if resistant parasites to ACT are in circulation in the study area, hence helping to mitigate the problem before much spread.

## Methods

### Study area

**Fig. 1:**
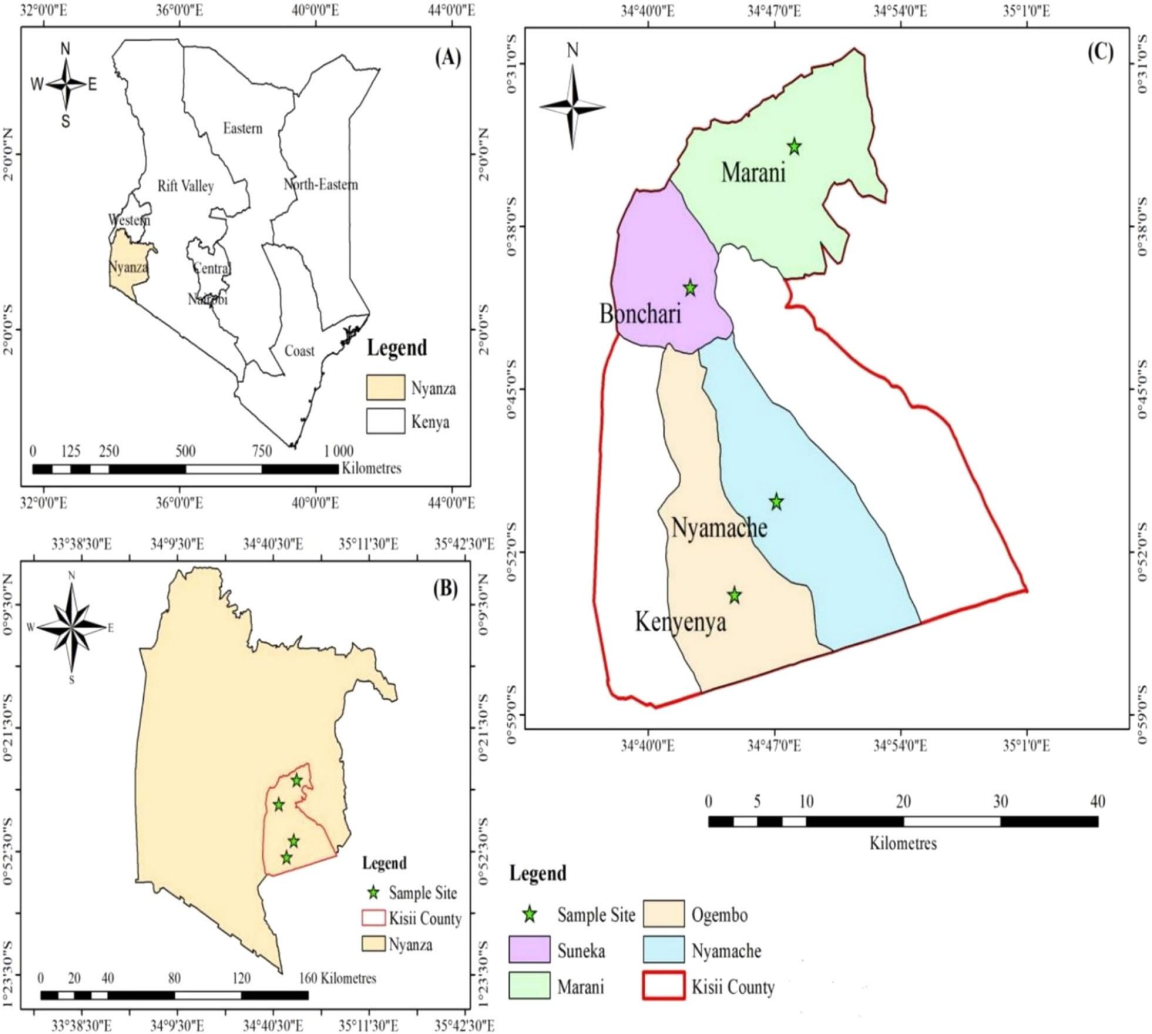
A map showing the study area. **A** Shows the country Kenya where Kisii County is located. **B** Shows Nyanza region where Kisii County is located and **C** shows different sub-counties of Kisii County where sampling was conducted (Map drawn by author)

This study was conducted in Kisii County, Kenya in the year 2021, during the months of February to June. The county has nine sub-counties. The county is located approximately 306 kms from the capital city, Nairobi. It lies at latitude: (0.41°) South**,** longitude: (34.46°) East. According to the 2019 Kenya population and housing census, the county population size is 1,266,660 persons [16]. The main economic activity is agriculture. The county is characterized by hilly topography interspersed with ridges and valleys. The county is characterized by seasonal and permanent rivers which flow into Lake Victoria. The county exhibits a highland equatorial climate with an average rainfall of 1500 mm/year. The average temperature range is between 21 and 30 °C. Most of the population lives in rural areas, residing in local houses. The county health system consists of government and private-based health facilities. The government sector has one teaching and referral hospital (KTRH), which serves as a regional reference hospital and a teaching hospital for Kisii University Medical School. This county contains 14 sub-county hospitals. The county also contains 84 dispensaries, 28 health centres and 32 community health units, which serve as centres for minor health cases [17]. The county records three rain seasons namely; April–May, August–September and November–December. The main killer disease is malaria. The main malaria intervention approaches used to combat malaria in this region include proper case management with anti-malarial drugs, such as artemisinin-based combinations, intermittent prophylaxis during pregnancy (IPTp) and the use of mosquito nets. The current drug of choice for treating uncomplicated malaria is artemether-lumefantrine. Diagnosis and treatment services of malaria are available in all government health facilities and a few private facilities. The current study was conducted in hospitals selected from 4 sub-counties (Kenyenya, Marani, Bonchari,Nyamache) of Kisii County (Fig. 1). A molecular study was conducted at the Molecular Biology and Immunology Laboratory, School of Health Sciences, Makerere University, Kampala, Uganda.

### Study design, study population and specimen collection

This was a cross-sectional health point prospective study. The study enrolled participants who before the start of the study were presented with malaria clinical characteristics and had resided in Kisii County for at least the last six months. However, the study excluded those who were reluctant to give consent to the study. In addition, participants with febrile clinical illnesses initiated by pathogens other than malaria were excluded from the study. Before collecting blood specimen, a complete medical examination and demographic information were obtained. All patients who had been suspected of having malaria infection by having a fever (≥ 38 °C) or having a history of fever in the past 24 h, were confirmed for the presence of *P. falciparum* using microscopy (as a confirmatory test). Briefly, thick and thin blood smears were stained with 2% Giemsa for 30 min. A smear was considered negative if no parasites were observed after examination under 100 high-powered fields. Thin smears were fixed in methanol before Giemsa-staining. Blood samples were collected by obtaining 1 ml of venous blood for the participants older than 2 years. 100 μL finger-pricked blood samples were collected in the case of children below 2 years of age. This procedure was repeated during the consequent follow-up visits. The blood spots were made on chromatography filter paper (ET31CHR; Whatman Limited, Kent, UK) and labelled with the participant identification number. Malaria-positive participants were followed up for a period of 28 days by evaluating clinical and parasitological parameters on days 1, 3, 7, 14, and 28, respectively, after AL treatment initiation. Finger pricks for follow-up were taken on days 1, 3, 7, 14 and 28 to check for the presence of *P. falciparum* [18].

### Characterization of merozoite surface protein-2 (MSP-2)

To distinguish between recrudescence and re-infection, 4 drops of blood from malaria-positive patients were collected on filter paper on day zero before treatment, and on any day of recurrent *P. falciparum* malaria. Molecular analysis was conducted following the previously described method [19], with slight modifications. Briefly, blood spotted filter papers were soaked for 24 h in 1 mL of 0.5% saponin-1 phosphate buffered saline. The mixture was washed twice in 1-mL PBS and boiled for 8 min in 100 mL PCR-grade water to release DNA from the cells. To elute the extracted DNA, 150 µL Buffer AE was added to each well using a multichannel pipette and incubated for 1 min at room temperature. This setup was then centrifuged at 2608 RCF for 8 min. DNA was recovered and stored at -80 °C. Nested PCR was performed on the extracted DNA for subsequent genotyping of *P. falciparum* polymorphic gene loci encoding Merozoite surface protein 2 (MSP-2) using the method described by [20]. A master mix was prepared according to manufacturer instructions (New England Bio Labs, Massachusetts, USA). 24 µL of the Master Mix was added to the PCR 96 well plate and 25 µL of the master mix was also added to the negative PCR control. The plates were sealed using a thermo-seal plate sealer and placed in the PCR thermo-cycler. Amplification was then performed under the following conditions; denaturation (94 °C), annealing (55 °C), and extension (72 °C). Amplification was confirmed by running the nested PCR product together with a DNA ladder on the QIAxcel capillary electrophoresis. The result was classified as recrudescence if at least one identical MSP2 allele was detected in both ACT pre-treatment and ACT post-treatment blood samples. Blood samples where MSP2 alleles did not match ACT pre- and ACT post-treatment were classified as new infections. Any sample, which failed to amplify was classified as undetermined. Blood samples, which showed recrudescence of parasites during any follow up day were further genotyped for *P. falciparum k13* resistance markers. The primers used in this protocol are shown in Table 1.

**Table 1:** Showing Merozoite Surface Proteins-2 (MSP-2) Amplification primers

Primer name	Sequence (5′ → 3′)	Purpose
MSP-2(1)	ATGAAGGTAATTAAAACATTGTCTATTATA	External forward primer
MSP-2(4)	ATATGGCAAAAGATAAAACAAGTGTTGCTG	External reverse primer
MSP-2(A1)	CAGAAAGTAAGCCTTCTACTGG	Internal forward primer (IC3D7)
MSP-2(A2)	GATTTGTTTCGGCATTATTATGA	Internal reverse primer (IC3D7)
MSP-2(B1)	CAAATGAAGGTTCTAATACTA	External forward primer (FC27)
MSP-2(B2)	GCTTTGGGTCCTTCTTCAGTTGATTC	Internal reverse primer (FC27)

### Sequencing of *k13*-propeller genes

DNA was extracted from blood spotted filter papers by using the chelex suspension method described by [20], with slight modifications. Blood spotted filter papers were soaked for 24 h in 1 mL of 0.5% saponin-1 phosphate buffered saline. The mixture was washed 2 times in 1-mL PBS and boiled for 8 min in 100 mL PCR-grade water. To elute the extracted DNA, 150 µL Buffer AE was added to each well using a multichannel pipette and incubated for 1 min at room temperature. This setup was centrifuged at 2608 RCF for 8 min to recover the DNA and stored at -30 °C. *K13*-propeller genes were amplified by the nested PCR protocol described previously [21] by using the primers listed in Table 1. For the first round of PCR, 0.5-mL DNA was amplified with 10 mL PCR Mix (1.25 U/mL, Taq DNA Polymerase, 0.4 mMdNTP Mixture, PCR buffer, and 4 mM Mg2þ), 0.5 mL forward primer (10 mM), 0.5 mL reverse primer (10 mM), and sterile ultrapure water to a final volume of 20 mL. For the second round of PCR, 0.5 mL primary PCR products were amplified with a 40 mL reaction system, including 20 mL PCR Mix, 1.0 mL forward primer (10 mM), 1.0 mL reverse primer (10 mM), and H2O. The amplification conditions were maintained at 95 °C for 3 min; followed by 30 cycles (95 °C for 30 s, 55 °C for 30 s, 72 °C for 30 s); 72 °C. For 5 min; then stored at 12 °C. Sequencing was done using the Sanger method described by [22], with slight modifications. The second round PCR products were purified by using a jet quick PCR product purification kit. 5 μL of the purified second round PCR products were then run on 1.0% (w/v) agarose gel stained with 0.05 μg/mL of Ethidium Bromide (Sigma Aldrich, USA) to counter-check for the presence and concentration of PCR products. This was followed by Bi-directional cycle sequencing of *K13* using the second round K133 Forward and K132 Reverse PCR primers (Eurofins Genomics, Germany), using the Big Dye terminator v3.1 cycle sequencing kit (Applied Biosystems, USA). Cycle sequencing PCR was performed in a total reaction volume of 20 μL. 6.0 μL of the Big Dye terminator v3.1 5X sequencing buffer (Applied Biosystems, USA). This was accomplished by mixing the above total reaction volume with; 2.0 μL Big Dye terminator v3.1 (Applied Biosystems, USA), 1.0uL of 10 ng/μL (K133 forward or K132 Reverse) primers and 6.0 μL of nuclease-free water. Finally, 5.0 μL of 5.0 ng/μL of the purified second round PCR products were then added to make up the total volume. The following sequencing conditions were used. One cycle of polymerase activation at 96 °C for 60 s followed by 35 cycles of; denaturation at 96 °C for 10 s, annealing at 53 °C for 30 s and extension at 60 °C for 300 s (Gene Amp 9700 PCR system, USA). The amplified products were then stored at 4 °C until the next step of extension. The extension PCR products were then followed by purification using Dye Ex 2.0 spin Kit (QIAGEN, Maryland, USA). Subsequently, 5.0 μL of the purified cycle sequencing PCR products were then mixed with 5.0 μL of De-ionized formamide (Sigma Aldrich, USA) and then loaded in the 3130 genetic analyzer (Applied Bio systems, USA). Finally, the products the products were bi-sequenced with POP-7™ (Applied Bio systems, USA) as a sequencing Polymer. The primers used in this protocol are shown in Table 2.

**Table 2:** Showing primers and gene fragment for evaluating artemisinin resistant *P. falciparum* markers

Gene fragment	PCR primer sequence	Mutations analyzed
*k13*-propeller (1st round PCR)	5′_CGGAGTGACCAAATCTGGGA-3 5′_GGGAATCTGGTGGTAACAGC-3	T474I, M476I, A481V, Y493H, T508N, P527T, G533S, N537I, R539T, I543T, P553L, R561H, V568G, P574L, A578S, and C580Y
*k13*-propeller (2nd round PCR)	5′_TCAACAATGCTGGCGTATGTG-3 5′_TGATTAAG GTAATTAAAAGCTGCTCC-3	T474I, M476I, A481V, Y493H, T508N, P527T, G533S, N537I, R539T, I543T, P553L, R561H, V568G, P574L, A578S, and C580Y

### Sequence data analysis

The DNA sequences were analysed using the sequence analysis software 5 and then blasted on to the NCBI sequence database to confirm the *k13* propeller gene sequence identity by using the Basic Local Alignment Search Tool (BLAST) at http://blast.ncbi.nlm.nih.gov/Blast.cgi. The sequences were exported to bio edit sequence alignment editor 7.2.5 for manual editing and then in to MEGA 5 software version 5.10 for detection of polymorphism using the PF3D7_1343700 and *K13*-propeller gene sequences present in the NCBI database were used as the reference sequence. Additional single-nucleotide polymorphism (SNPs) analysis within the *K13* propeller gene was performed using the DnaSP software version 5.10.01. To assess the selection pressure in *P. falciparum* parasite population in Kisii County, Tajima’ D statistic and Fu & Li’s D test in DnaSP software 5.10.01 were used. In this analysis, the study evaluated whether the *P. falciparum k13* propeller domain sequence data show evidence of deviation from the neutrality theory of molecular evolution. The analysis was done using commands in the DnaSP software. In the DnaSP software, the probability of Tajima’s D and Fu & Li’s D are estimated by simulation. The test uses information on the frequency of mutations (allelic variation) [23]. Tajima’s D and Fu & Li’s D test is based on the fact that under the neutral model, estimates of the number of polymorphic sites and the average number of nucleotide differences are correlated. The critical values (Tajima’s D and Fu & Li’s D) obtained were used in interpreting the findings under the neutrality assumption [24].

### Tajima’s D simulation

$$\frac{{{\text{D}} = {\text{n}} - {\text{s}}/{\text{d1}}}}{{\sqrt {{\text{Var }}\left( {\pi - {\text{S}} = {\text{a1 }}\raise.5ex\hbox{$\scriptstyle 1$}\kern-.1em/ \kern-.15em\lower.25ex\hbox{$\scriptstyle 2$} {\text{ p}}} \right)} }}$$

where, π, Mean pairwise differences; S, Number of segregating sites; p, total number of mutations.

### Fu and Li’s D simulations

$${\text{D}} = {\text{ }}\raise.5ex\hbox{$\scriptstyle 1$}\kern-.1em/ \kern-.15em\lower.25ex\hbox{$\scriptstyle 4$} {\text{ S}} - {\text{a1}} \cap {\text{e }} - {\text{a1}} \cap {\text{e p}}$$

where, ∩ e, Expected number of derived mutations that are present only once in the sample; S, Number of segregating sites

$${\text{a1}} = {\text{Xn}}\raise.5ex\hbox{$\scriptstyle 1$}\kern-.1em/ \kern-.15em\lower.25ex\hbox{$\scriptstyle 4$} {\text{1 i}}\raise.5ex\hbox{$\scriptstyle 1$}\kern-.1em/ \kern-.15em\lower.25ex\hbox{$\scriptstyle 4$} {\text{1 1}} = {\text{i}}$$

### Ethical consideration

Ethical approval was sought from the University of East Africa, Baraton Institutional Review Board (UEAB/REC/4/2/2021, research permit was issued by Kenya National Commission for Science, Technology and Innovation (NACOSTI) License No: NACOSTI/P/21/8974 and Kisii County government (DTR/4/27). The guidelines outlined in the Declaration of Helsinki were followed as follows; written informed consent was obtained from the adult participants. The consent of those below 18 years of age was provided by the parents or the care-givers. Permission for conducting this study was granted by different sub-counties before starting the study. Moreover, participation was voluntary. The participants were coded instead of reflecting their names to maintain confidentiality. Participants who were malaria positive were given antimalarial treatment according to the WHO regulations and they were reimbursed for the travel cost, lost earnings and food expenses. Participants were respected in relation to their right of their cultural beliefs and rights. Participants were allowed to withdraw from the research without any condition. Approval from local leaders was obtained before beginning the study. Since this study was conducted during the COVID-19 pandemic, standard operating procedures were followed as stipulated by the WHO.

## Results

### Base line characteristics of the participants

A total of 275 participants were recruited in 2021 during the months of February to June. More female (60.0%) participants were enrolled compared to males (40%). The mean age of the recruited participants was 27.60 ± 0.92 years. 69% (189.75 ± 0.25) of the participants were adults, with a nearly equal proportion between males and females. 84% (231.0 ± 0.84) of the participants completed the efficacy profiling on AL from day 0 to day 28. The temperature recorded at enrollment (day 0) varied across the sites, with Kenyenya recording 37.6 °C ± 1.1, Marani recording 37.5 °C ± 1.1, Bonchari recording 37.8 °C ± 1.1 and Nyamache recording 37.3 °C ± 1.1 respectively. However, the temperatures recorded during 28 days of study did not vary across the sites. The geometric mean parasite density (asexual parasites/µL) was significantly higher at Bonchari, having recorded 13,531 (9,242–15,603), (*p* = 0.047) compared to the other sites; patients enrolled at Nyamache had the lowest parasitaemia. The mean weight and median age range varied across all sub-counties (Table 3).

**Table 3:** Base line characteristics of participants enrolled in therapeutic efficacy study at 4 sentinel sites of Kisii County

Variables	Kenyenya n (%) (n = 69)	Marani n (%) (n = 69)	Bonchari n (%) (n = 69)	Nyamache n (%)(n = 68)	*P* value
*Sub-Counties*					
Weight (kg), mean (SD)	44.8 ± 7.3	39.9 ± 15.5	36.4 ± 9.7	38.4 ± 8.5	0.15
Gender (male), n (%)	31 (44.9)	28 (40.5)	31 (44.9)	20 (28.9)	0.38
Gender (female), n (%)	38 (55.0)	41 (59.4)	38 (55.0)	48 (71.1)	0.51
Body temperature on day 0, °C, mean (SD)	37.6 °C ± 1.1	37.5 °C ± 1.1	37.8 °C ± 1.1	37.3 °C ± 1.1	0.002*
Asexual Parasites /µL on day 0* (95% CI)	11,4301 (7,256–12,785)	12,403 (8,242–13,565)	13,531 (9,242–15,603)	9,221 (7,308–11,252)	0.047*
Median age in years (males)	34.5	38.5	42.5	41.5	0.10
Age range in years (males)	(4–62)	(3–82)	(5–69)	(2–75)	0.172
Median age in years (females)	31.5	28.5	27.5	30.5	0.75
Age range in years (females)	(5–74)	(3–75)	(5–80)	(2–65)	0.125

°C: degree Celsius; Temperature of ≥ 37.5 °C or history of fever during the previous 24 h. Parasitaemia*: geometric mean parasite density (asexual parasites/µL); n: number of patients; SD: standard deviation; 95% CI: 95% confidence interval; *p < 0.05, the mean was significantly different

### Recrudescence molecular outcome

**Fig. 2:**
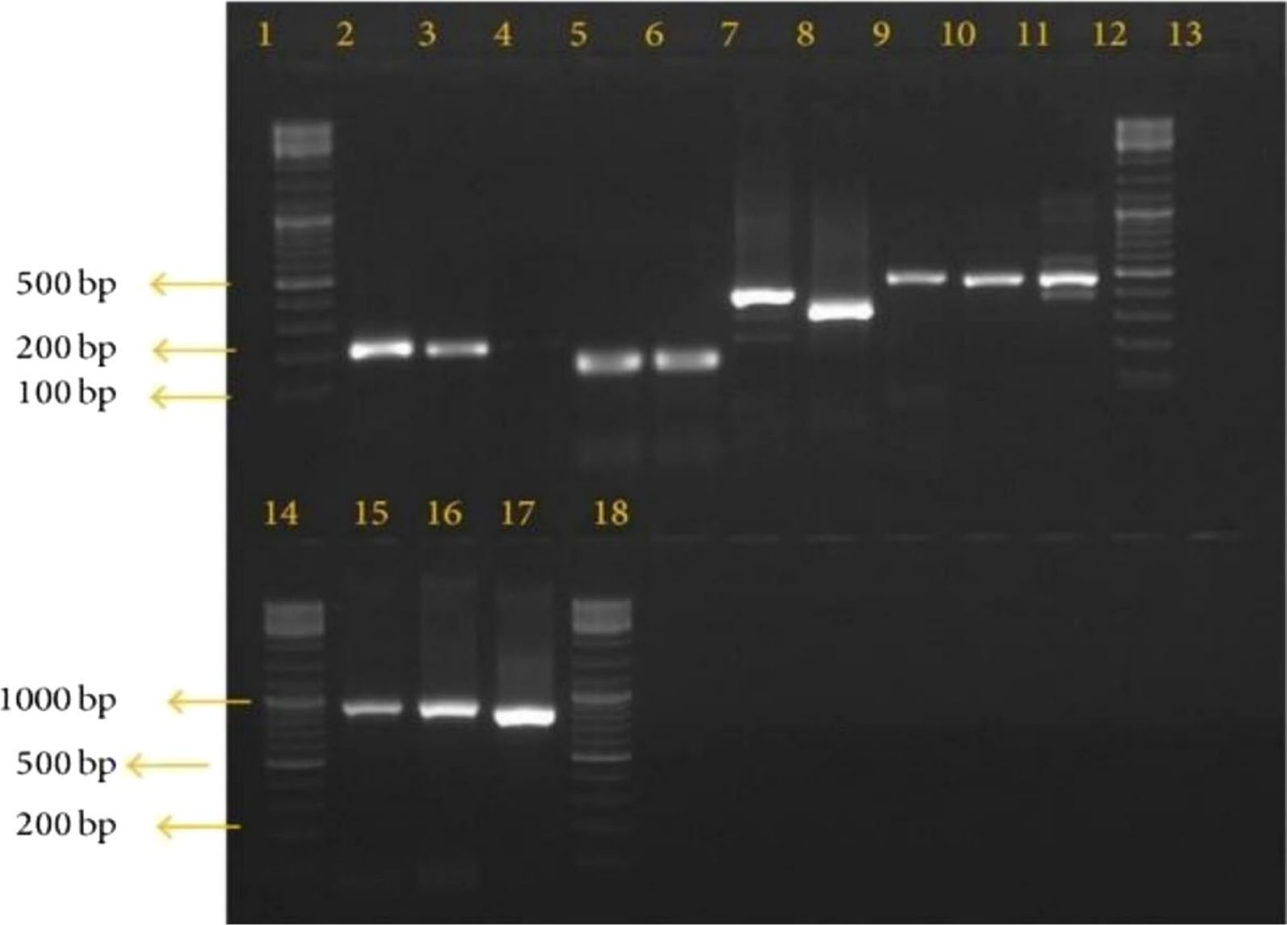
Gel image showing the amplification of *P. falciparum msp2* of recurrent samples. Bands 2,3,4,5,6,7,8,9,10 and 11 shows positive msp2 allelic family. Band13 is the negative control, and lanes 1, 12, 14, and 18 shows 100 bp Molecular Weight DNA ladder (New England BioLabs, Massachusetts, USA)

After a complete follow-up of the participants, 13/231 (5.6%) had *P. falciparum* on day 28. For those samples with the occurrence of parasites on day 28 after treatment had the following PCR; 77% of the samples had bands on both day 0 and day 28, hence were classified as recrudescence. About 23% of the samples had no bands on both days 0 and the respective days of parasite recurrence, hence classified as new infections (Fig. 2). The 77% of samples showing recrudescence in this study were stored for further *P. falciparum kelch13* sequencing to evaluate the presence of any mutational polymorphisms conferring ACT resistance.

### PCR amplification of *Pfkelch13* genes

**Fig. 3:**
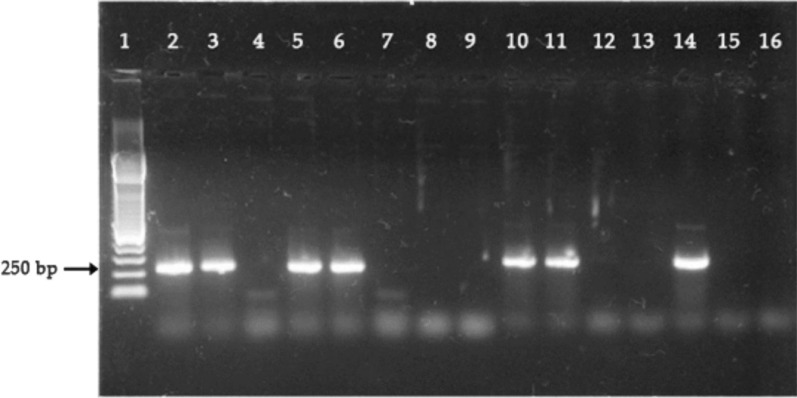
Positive PCR products detected using Agarose Gel Electrophoresis. Well 1,2,15, and 16 contain 250 bp DNA ladder, well 8 and 9 contain positive control *(PF3D7_1343700*). The rest of the well contains samples. Samples 3,5,6,10,11 and 14 are positive for *Pfkelch13* mutations while samples 4,7,12 and 13 are negative for *Pfkelch13* mutations. The expected amplicon size was approximately 800 bp

A total of 13 blood samples confirmed for day 28 recrudescence using microscopy correction were processed for *Pfkelch13* PCR amplifications. Parasites DNA of 5 (38%) samples were successfully amplified as shown in Fig. 3 and Table 4 by using nested PCR reaction for the *k13*-propeller gene mutations and hence were sequenced for mutations.

**Table 4:** No of cases of 28 day parasite recrudescence and *Pfkelch13* amplified genes

Sub County	No. of samples with recrudescence (n / %)	*PfK13* amplified (n/%)
Marani	3 (23)	1 (17)
Bonchari	2 (15)	2 (33)
Kenyenya	3 (23)	1 (17)
Nyamache	5 (39)	2 (33)
Total	13 (100)	6 (100)

### Frequencies of *k13* propeller mutations

*K13* propeller single-nucleotide polymorphisms were compared with the *3D7* reference strain (PF3D7-1343700). *K13*-propeller non-synonymous polymorphisms recorded in the current study include R539T, N458Y, R561H, N431S, and A671V. All mutations with an exception of R561H were reported in 1 sample, while R561H mutation was reported in 2 samples, respectively. However, mutation on *K13* propeller gene was not detected at positions 580, 578, 574, 568, 553, 543, 539, 537, 533, 527, 508, 493, 481, 476, and 474, respectively. A detailed analysis of the samples is shown in Table 5. In total, there were six (6) polymorphic sites identified in the 13 samples analysed.

**Table 5:** Frequencies of *K13* –propeller mutations

Sample ID	No. of codon	Wild type A.A	Mutant type A.A	Genetic change	Type of mutation
K13-MA-007	R539T	Arg	His	GCT → TCT	Non-synonymous
K13-BO-011	N458Y	Arg	Val	TCT → TCG	Non-synonymous
K13-BO-053	R561H	Arg	Ser	GCA → TCA	Non-synonymous
K13-NYA-040	N431S	Tyr	Glx	ATG → GTG	Non-synonymous
K13-NYA-030	A671V	Asn	Tyr	TTA → ATA	Non-synonymous

### Haplotype diversity

The mutations in the samples analysed were unselectively neutral, as shown by the negative Tajima’s D statistic (− 1.72305) and Fu & Li’s D test (− 1.74248). There was no significant haplotype/gene diversity (*P* = 0.305) with a variance in the diversity of 0.00363 and a standard deviation of 0.078 (Table 6).

**Table 6:** Haplotype diversity of the clinical isolate

*K13* analyzed Variables	Sub-Counties				
	Nyamache	Kenyenya	Marani	Bonchari	Overall
Sample size	5	3	3	2	13
Number of haplotypes(h)	2	1	1	2	6
Number of nucleotide sites analyzed	671,458	561	539	561,458	671,458,561,539
Haplotype(gene) diversity (hd)	0.312	0.324	0.315	0.302	0.305
Standard deviation of haplotype diversity	0.082	0.73	0.72	0.89	0.078
Nucleotide diversity	0.00242	0.00115	0.00214	0.00131	0.000129
Variance of haplotype diversity	0.00724	0.01342	0.00563	0.00418	0.00363
Tajima’s D statistic	− .0.78621	− 1.7346	− 0.3827	− 1.8317	− 1.74248 (Not significant; *P* < 0.05)
*Fu* and *Li*’s D test statistic	− .0.34123	− 1.7346	− 0.3827	− 1.8317	− .1.74248 (Not significant; *P* < 0.05)
No of polymorphic sites	2	1	1	2	6

## Discussion

Resistance occurs as a result of mutations in the target points in the parasite. Limited countries across South East Asia and malaria-endemic Africa have revealed evidence of low frequency ART-resistance linked mutations, with an initial indication of indigenous *Pfk13* mutations in the East Africa region, speculating that the threat of independent acquisition of resistance should be taken seriously [25]. There is a critical need for augmented, uniform and prospective anti-malarial resistance molecular surveillance across Africa. Investigation of the association between *P. falciparum* mutations and reduced susceptibility to ACT through genome-wide association studies (GWAS) and gene manipulation studies, have shown a relationship between mutations in *k13* and increased parasite survival in the in vitro conditions [26].

Mutations in the kelch13 gene has been identified as ACT resistant molecular markers. Different mutations have been previously reported in Asia, America and Africa continents, with more prevalence recorded in the Asian continent [27]. The evolution and spread of mutant *P. falciparum k13*-mediated artemisinin (ART) resistance has led to extensive treatment failures all over Southeast Asia [28]. *Plasmodium falciparum* resistance to artemisinin derivatives has been reported across Southeast Asia (SEA), having first confirmed a decade ago in western Cambodia [29–31].

The present study has reported the presence of *Pfk13* polymorphisms at different loci. The mutations detected here include R561H, R539T, N458Y, N431S and A671V. However, the frequencies of the mutations were low compared to those witnessed in ACT resistant geographical locations. R561H Single Nucleotide Polymorphism hereby reported in one sample from Bonchari and Marani sub-counties has previously been associated with reduced parasite clearance in South East Asia [32]. Moreover this mutation has been previously reported in Rwanda [33] and Tanzania [34], countries located in East Africa, thus raising concern on the probability of importation of ACT resistant parasites as a result of human movements. Despite the fact, the mutation was only detected in one sample, this data emphasize the threat of the R561H mutation spreading in Kenya. Particularly, by comparing with the past great quantity of R561H mutation across the Myanmar and Thailand [35], the presence of the R561H variant in the study area points to the risk of an emergence of ACT resistance.

Single Nucleotide Polymorphism (SNP) observed in locus R539T in the current study has also been reported in Kenya and Senegal. R539T was reported from a study conducted in Mbita district, Kenya [36]. This mutation was highly associated with in vivo delayed parasite clearance among the patients from Mbita district. This mutation has been previously associated with ACT resistance in South East Asia, thus raising much concern on the possibility of resistance spread.

The mutation reported in the N458Y locus has also been reported elsewhere in Africa and other South Eastern Asian countries. Previously this mutation has been used as a validated candidate of resistance since it is associated with poor drug response of *P. falciparum* to ACT [37]. The low frequencies of mutations associated with ACT resistance reported here in comparison to South East Asia which has reported high frequency may be due to the fact that ACT usage in SEA started a long time ago, compared to Kenya which adopted ACT usage in 2004 [38].

Biological factors such as immunity may be contributing in influencing the development of resistant phenotypes. It has been previously hypothesized that acquired immunity against malaria parasites eliminates the parasites independent of anti-malarial agents [39]. Previously documented report from a multinational study has ascertained that naturally acquired immunity to *P. falciparum* differs from one population to another. The study indicated that immunity was lowest in regions with high prevalence of *kelch13* mutations and slow parasite clearance phenotype. Thus suggesting that host immunity contributes to the clearance of drug-resistant parasites [40]. Immunity is high in those areas with high malaria transmission compared to those in low malaria transmission. SEA is a low malaria transmission area, hence enhancing low acquired immunity in the population. Acquired immunity increases the clearance of ACT resistant *P. falciparum* parasites. Naturally acquired immunity to malaria develops after repeated exposure to parasites, and is acquired faster in high- compared to low-transmission areas.

The current study is the first report on the mutations associated with N431S and A671V, respectively. This is in tandem with other studies which have reported new mutations in *P. falciparum* clinical isolates [41]. More than 200 non-synonymous mutations have been recognized in the K13 protein from *P. falciparum* [42]. However, fifty *Pfk13* mutations have been reported previously to be associated with ACT resistance in South East Asia of which nine have been confirmed as resistant candidate while eleven are potentials for ACT resistance. The other thirty *k13* mutations have been reported from various locations in South Asia, however, they are not consistent with the clinical findings on ART resistance. Out of these documented mutations, only 11 have been authenticated as candidates for ART resistance under the ex vivo conditions. The validated mutations present in the propeller domain conferring ART resistance, includes F446I, N458Y, M476I, Y493H, R539T, I543T, P553L, R561H, P574L, C580Y and A675V [43].

Surprisingly, previous studies have reported new non-validated mutations, which were present in patients who had poor recovery after treatment with ACT [44]. This raises concern about whether they have some roles they play in conferring resistance, and should hence be considered in the future as potential molecular markers of ACT resistance. A mutation at codon A675V has been reported in Rwanda [45] and Uganda [46]. The same mutation at codon A675V had also previously been reported in SEA [47], an epicentre of the emergence and spread of ACT resistance.

The circulation of *k13* mutations have also been previously reported in Kenya, but with limited studies [48]. A previous study conducted in different malaria transmission areas of Kenya viz; Marigati, Kombewa, Kisumu, Kisii, Kericho and Malindi to ascertain the prevalence of *k13* mutation during the pre-ACT and post-ACT periods, reported different polymorphisms at different locus [49]. The A578S and the V568G mutations reported in SEA were found in both pre-ACT and post-ACT parasites. D584Y and R539K mutations were found only in post-ACT parasites. These mutations were also previously reported from clinical isolates from South East Asia [50], raising the question of the possibility of mediating resistance. The N585K mutation was described for the first time in this previous study among the post-ACT parasites, and it was the most prevalent mutation at a frequency of 5.2%. However, the prevalence and type of mutations varied across the malaria ecological zones and between the pre-and post-ACT periods. This study reported A578S in post-ACT parasites in two different study sites, Kombewa (4.3%) and Kisii (2.1%). Kombewa is situated in the holo-endemic lake region and Kisii is located in the highland epidemic region. The N585K allele was reported in only the post-ACTs era in the study areas, with the highest prevalence in Kombewa (10.6%) and Kisumu (9.8%). This mutation witnessed here might be under pressure for evolution through anti-malarial drugs since the use of artemether-lumefantrine is high in Kombewa and Kisumu due to high malaria transmission [51].

Another study conducted at 4 islands in the Lake Victoria basin (Kibuogi, Ngodhe, Takawiri, and Mfangano) and the mainland of Mbita in Kenya reported different mutant alleles in the *k13* gene, with C580Y, Y493H and R539T being the most prevalent and significantly associated with in vivo delayed parasite clearance [52]. However, a new mutation of A578S was detected at Mfangano Island for two consecutive seasons. This mutation is closely related to the single nucleotide polymorphism C580Y detected from Cambodia, indicated to be conferring ACT resistance [53]. Other mutations reported in this study included M442V, N554S, A569S, C439C, S477S, Y500Y, N531N and G538G. These mutations have not been previously associated with ACT resistance.

A previous study on the Kenyan coast has reported limited *P. falciparum k13* artemisinin resistance-conferring mutations over a 24-Year Analysis. The K189T mutation was the only polymorphism maintained at frequencies of 10%, while the rest of the observed alleles were rare, including codon A578S, with frequencies barely reaching 2% [54].

A report by the WHO in 2021 has documented a 30-fold increase in the use of ACT globally between 2006 and 2013 [55]. Thus, the augmented usage of artemisinin agents is expected to increase drug pressure, leading to resistance development. Consequently, irrational usage of ACT coupled with the use of substandard drugs in developing countries such as Kenya, may exacerbate the risk of resistance development. Bearing in mind that previous resistance to anti-malarial agents was first detected in South East Asia and then later spread to Africa, it is possible that the artemisinin resistance documented in Cambodia may also spread through Myanmar via India to Africa by following the previous patterns [56]. This is likely to occur due to the increased international travel and migration, especially because Kenya serves as a transition point for travellers from Asia to Africa and South America.

After testing the genetic departures of nucleotide variability patterns of the sequence products from neutral expectations, the isolates from the current studies showed evidence of positive selection as highlighted by the negative values of the tests (Tajima’s D =  − 1.72305; Fu and Li’s D of − 1.74248). Negative values suggest that the genes present in the parasites had experienced nonrandom processes leading to genetic selection. Additionally, since parasite samples used in this study were obtained after the implementation of ACT, these nonrandom processes are related to ACT pressure. The direction of selection statistic was positive, implying an excess of non-synonymous polymorphisms, suggesting that slightly deleterious alleles were circulating in the parasites. Previous study has documented that indigenous populations and ecological level courses, such as drug pressure serve as essential mediators of resistance acquisition in the population [57]. The current study was unable to establish if the *k13* gene mutations detected in the *P. falciparum* clinical isolates from Kisii County resulted from local emergence because of ecological and population-level processes or through transfer because of global human travel or local emergence. In contrast to Africa and Kenya, where artemisinin agents are commonly used in the form of combinations, studies have indicated that more than 78% of artemisinin in South East Asia is used as monotherapy [58].

It was recently demonstrated that *k13* mutation outside the propeller domain can be linked with ART-resistance [59]. This gene encodes a 726-amino acid protein (PfK13) comprising of three highly conserved domains: a coiled-coil-containing domain, a BTB/POZ domain, and a Kelch-repeat beta-propeller domain [60]. It will be very informative for surveillance of ART-resistance emergence to extend *k13* sequencing to the BTB/POZ domain of the protein. The accumulation of data from Kenya will increase the understanding of the association between the *k13* gene and artemisinin resistance. Unfortunately, most of the molecular drug surveillance conducted in Kenya was performed in western and coastal regions. Thus no clear picture of the molecular data is available.

## Conclusions

The current study has reported low circulation of the previously validated *Pfk13*-resistance markers, with two new mutations which have not previously been reported globally. However, there were limited polymorphisms for the previously reported *k13* mutations validated as resistance markers. Due to insufficient evidence, the study concludes that artemisinin resistance is yet to be confirmed in Kisii County. Moreover, the new single-nucleotide polymorphism mutations detected in this study need to be characterized further to ascertain their role in conferring ACT resistance. In addition, molecular surveillance of drug resistance needs to be scaled up in Kenya to provide regular updates on the possibilities of the emergence and spread of ACT resistance for future malaria containment.

## Data Availability

The sequences have been deposited in NCBI with bio-project number PRJNA885380 and accession numbers SAMN31087430, SAMN31087431, SAMN31087432, SAMN31087433, and SAMN31087434 for Marani, Nyamache, and Bonchari samples, respectively.
